# Role of Amino Acid Transporter SNAT1/SLC38A1 in Human Melanoma

**DOI:** 10.3390/cancers14092151

**Published:** 2022-04-26

**Authors:** Ines Böhme-Schäfer, Sandra Lörentz, Anja Katrin Bosserhoff

**Affiliations:** Department of Biochemistry and Molecular Medicine, Institute of Biochemistry, Friedrich-Alexander Universität Erlangen-Nürnberg (FAU), Fahrstraße 17, 91054 Erlangen, Germany; ines.boehme@fau.de (I.B.-S.); sandra.loerentz@fau.de (S.L.)

**Keywords:** tumor metabolism, melanoma, amino acid transporter

## Abstract

**Simple Summary:**

Malignant melanoma originates from melanocytes. Due to its high metastatic potential and its increasing incidence, it is one of the most aggressive types of cancer. Cancer cells generally exhibit an elevated metabolism, consequently adapting their expression of transport proteins to meet the increased demand of nutrients, such as amino acids. The aim of this study was to analyze the expression and function of the amino acid transporter SNAT1 in human melanoma. In addition, we wanted to determine its role in development and progression of malignant melanoma. We revealed that SNAT1 is overexpressed in melanoma tissue samples, as well as primary and metastatic cell lines. Moreover, we were able to show that SNAT1 plays an important role in forcing proliferation, colony formation, migration and invasion, and inhibiting senescence of melanoma cells. Amino acid transporters like SNAT1 are therefore promising targets for the development of novel therapeutic strategies against melanoma.

**Abstract:**

The tumor metabolism is an important driver of cancer cell survival and growth, as rapidly dividing tumor cells exhibit a high demand for energetic sources and must adapt to microenvironmental changes. Therefore, metabolic reprogramming of cancer cells and the associated deregulation of nutrient transporters are a hallmark of cancer cells. Amino acids are essential for cancer cells to synthesize the necessary amount of protein, DNA, and RNA. Although cancer cells can synthesize glutamine de novo, most cancer cells show an increased uptake of glutamine from the tumor microenvironment. Especially SNAT1/SLC38A1, a member of the sodium neutral amino acid transporter (SNAT) family, plays an essential role during major net import of glutamine. In this study, we revealed a significant upregulation of SNAT1 expression in human melanoma tissue in comparison to healthy epidermis and an increased SNAT1 expression level in human melanoma cell lines when compared to normal human melanocytes (NHEMs). We demonstrated that functional inhibition of SNAT1 with α-(methylamino) isobutyric acid (MeAIB), as well as siRNA-mediated downregulation reduces cancer cell growth, cellular migration, invasion, and leads to induction of senescence in melanoma cells. Consequently, these results demonstrate that the amino acid transporter SNAT1 is essential for cancer growth, and indicates a potential target for cancer chemotherapy.

## 1. Introduction

In the last 50 years, the incidence of melanoma has increased significantly. Melanoma cells are very invasive and have a high metastatic potential already at early stages. Patients with metastatic melanoma (stage IV) exhibit a five-year survival rate of only 22.5% [1]. Despite major advances in targeted and immunotherapy in recent years, the emergence of therapy resistance demonstrates that there is still a great need for research to better understand the development and progression of melanoma.

Tumor cells can reprogram their cellular metabolism and upregulate transport proteins to acquire necessary nutrients and maintain tumor survival and growth. Whereas differentiated, non-dividing cells metabolize nutrients via the tricarboxylic acid cycle and the respiratory chain, resulting in the production of CO_2_ and H_2_O, rapidly proliferating cancer cells are able to change the metabolic pathways they depend on. For instance, melanoma cells upregulate the gene expression of glucose transporter isoform 1 and 3 (GLUT1/SLC2A1 and GLUT3/SLC2A3) to take up a high amount of glucose [2]. Under aerobic conditions, glucose is metabolized to pyruvate, which is mainly utilized for lactic acid production. This metabolic pathway of aerobic glycolysis is called Warburg effect [3]. The increased production and secretion of lactate and H^+^ into the extracellular environment causes an acidification of the tumor microenvironment, which triggers the development of a therapy-resistant and senescence-like phenotype in melanoma [3,4].

Besides the Warburg effect, glutaminolysis [5] is considered as another important hallmark of tumor cell metabolism. Although cancer cells have the capacity to synthesize glutamine (Gln) de novo, they exhibit an increased uptake of glutamine from the tumor microenvironment. Whereas the Na^+^-dependent neutral amino acid transporter ASCT2/SLC1A5 [6,7] and large amino acid transporters LAT1/SLC7A5 [8,9] and LAT2/SLC7A8 are described to accomplish exchange of amino acids to achieve amino acid homeostasis in cells, Na^+^-coupled neutral amino acid transporter SNAT1/SLC38A1 mediates net glutamine uptake for glutaminolysis [10].

SNAT1 is a member of the SNAT/SLC38 family. This SNAT family can be divided in group “A” and group “N” transporters. The “A”-transporters SNAT1/SLC38A1, SNAT2/SLC38A2, and SNAT4/SLC38A4 mediate the transport of a variety of small neutral amino acids, whereas the “N”-transporters SNAT3/SLC38A3, SNAT5/SLC38A5, and SNAT8/SLC38A8 mediate a more specific transport of glutamine, asparagine, and histidine, respectively [11]. Although SNAT1 is able to transport small neutral amino acids, it is described as the main glutamine loader [10]. Physiologically, SNAT1 is mainly expressed in testis, placenta, brain, endocrine tissues, respiratory system, and gastrointestinal tract (www.proteinatlas.org, accessed on 22 November 2021). The amino acids that are transported by SNAT1 serve as a carbon and nitrogen source for anabolism, but are also used for glutathione synthesis, which neutralizes reactive oxygen species (ROS). SNAT1 is also implicated in cellular signaling. It is a positive regulator of mTORC1 in healthy mouse neurons [12]. Additionally, SNAT1 overexpression increases the phosphorylation level of Akt (p-Akt) and mTOR (p-mTOR) in osteosarcoma [13], breast cancer [14], and hepatocellular carcinoma (HCC) [15].

Studies demonstrated that SNAT1 is overexpressed in colorectal cancer [16], breast cancer [14], HCC [17], gastric cancer [18], osteosarcoma [13], acute myeloid leukemia (AML) [19], and endometrial cancer [20]. SNAT1 overexpression is associated with increased tumor size, tumor invasion depth, migration, metastasis and proliferation, and poorer prognosis for patients [13,14,16,18,19,20]. Therefore, amino acid transporters such as SNAT1 that are overexpressed in cancer cells represent potential drug targets. Functional inhibition leads to nutrient deprivation in cancer cells, resulting in growth arrest and cell death, whereas normal cells may remain unaffected [21].

In this study, we observed that the Gln transporter SLC38A1/SNAT1 is highly expressed in human melanoma tissue, as well as many melanoma cell lines and investigated the functional role of SNAT1 in cell proliferation, migration, invasion, and senescence induction in melanoma.

## 2. Materials and Methods

### 2.1. Culturing of Melanoma Cell Lines

The human melanoma cell line Mel Juso (derived from primary cutaneous melanoma) was cultured in RPMI-1640 medium (Roswell Park Memorial Institute, Buffalo, NY, USA) with NaHCO_3_ and the cell lines Mel Im (isolated from melanoma metastases) and HEK293 were cultured in DMEM D6046 (Dulbecco’s Modified Eagle’s Medium, Sigma Life Science, St. Louis, MO, USA) low glucose. Both media were supplemented with penicillin (400 U/mL), streptomycin (50 g/mL), and 10% fetal calf serum (Sigma-Aldrich, München, Germany) at 37 °C in a humidified atmosphere containing 8% CO_2_ for Mel Im and Mel Juso and 5% CO_2_ for HEK293 (see Table 1 for an overview of all used cell lines). For functional inhibition of SNAT1, the amino acid analog MeAIB (Sigma-Aldrich, München, Germany) was used in 10 mM and 20 mM on melanoma cells. Control cells (CTR) were treated with equal amount of solvent ddH_2_O.

### 2.2. mRNA Expression Analysis

Total cellular RNA was isolated from melanoma cell lines using the Total RNA Kit I (Omega Bio-Tek, Norcross, GA, USA). Molecular generation of cDNA by reverse transcription was performed by using the SuperScript II Reverse Transcriptase Kit (Invitrogen). Relative mRNA expression was performed via quantitative real-time PCR (qRT-PCR) analysis on a LightCycler480 II device (Roche, Mannheim, Germany) with specific primers (see Table 2 for primer sequences) and normalized to β-actin as previously described [4].

### 2.3. Protein Analysis with Western Blotting

For protein isolation, cells were lysed in RIPA buffer (50 mM Tris/HCl pH 7.4, 150 mM NaCl, 1% N-P40, 0.1% SDS, 0.5% sodium deoxycholate, and protease inhibitor). An amount of 20 μg protein per lane were loaded and separated on 12.75% sodium dodecyl sulfate polyacrylamide gel electrophoresis gels. Proteins were transferred onto a polyvinylidene difluoride membrane and blocked in 3% bovine serum albumin (BSA) and 5% milk powder for 45 min. The membranes were incubated with primary antibodies: SNAT1/SLC38A1 (1:1000, HPA052272, Sigma-Aldrich, München, Germany) overnight at 4 °C and β-actin (1:5000, A5441, Sigma-Aldrich, München, Germany) in 5% BSA 1 h at room temperature followed by incubation with the secondary antibody (anti-rabbit–horseradish 1:2000 and anti-mouse–horseradish, 1:5000) for 1 h at room temperature. For protein staining, the Clarity^TM^ Western ECL Substrate kit (Bio-Rad Laboratories Inc., Hercules, CA, USA) was used, and protein signal intensity was detected by gel documentation system (Intas ECL Chemocam, Intas Science Imaging Instruments GmbH, Göttingen, Germany).

### 2.4. siRNA Transfection

For SNAT1 downregulation, 90,000 cells of melanoma cell lines Mel Im and Mel Juso were seeded into 6-well plates and were transiently transfected using Lipofectamine RNAiMAX reagent (Life Technologies, Darmstadt, Germany) with an siPool (containing approx. 30 specific siRNAs) against SNAT1 (functionally verified by siTOOLs Biotech, Planegg/Martinsried, Germany) or a negative control siPool. After 72 h incubation, the cells were transfected for additional 24 h and subsequently seeded for experimental analysis (total siPool incubation was 96 h). For siPools from this manufacturer, a previous study demonstrated low rate of off-target effects, as the global gene expression is not affected, and high reliability of siPools in comparison to siRNAs [22].

### 2.5. Immunohistochemical Analysis

Standard 5 µm sections of formalin-fixed and paraffin-embedded tissue blocks were used for immunohistochemical analysis of human tissue samples (tissue microarray), comprising specimens from benign nevi, primary melanoma, and melanoma metastases. Immunohistochemical staining was performed using an anti-SNAT1 antibody (SNAT1/SLC38A1, 1:100, HPA052272, Sigma-Aldrich, München, Germany). Sampling and handling of patient material were carried out in accordance with the ethical principles of the Declaration of Helsinki. The use of human tissue material had been approved by the local ethics committee of the University of Regensburg.

### 2.6. Immunofluorescence Staining

For immunofluorescence analysis, cells were fixed, permeabilized, and stained as described previously [4]. Images were acquired using the inverted microscope IX83 (Olympus Life Science, Tokyo, Japan). Microscope images were analyzed using Olympus CellSens Dimension Software 1.12 (Olympus Cooperation, Tokyo, Japan).

### 2.7. XTT Viability Assay

Cell proliferation and mitochondrial activity was analyzed using the Cell Proliferation Kit II (XTT) (Roche Diagnostics GmbH, Mannheim, Germany). MeAIB-Inhibitor treatment started 6 h after cell seeding. XTT analysis of siSNAT1-downregulated cells compared to control-transfected (siCTR) cells started 120 h after siPool transfection. XTT reagent was added 24, 48, 72, 96, 120, and 144 h after cell seeding to adherent cells according to the manufacturer’s instructions, and absorbance was measured with a CLARIOstar plate reader (BMG Labtech GmbH, Ortenberg, Germany) at 490 nm.

### 2.8. Analysis of Cell Proliferation

Real-time cell proliferation was measured using the xCELLigence System (Roche Diagnostics GmbH, Mannheim, Germany) and E-plates (ACEA Bioscience, San Diego, CA, USA), as described in [23].

### 2.9. Clonogenic Assay

To analyze attachment-dependent colony formation and growth of cancer cells, clonogenic assays were performed as described before [24].

### 2.10. Scratch Wound Healing Assay

The migratory behavior of cells was analyzed with the scratch wound healing assay. 300,000 melanoma cells were seeded into 6-well plates. After 24 h, the cell monolayer was scratched with a pipette tip in a definite area. Migration rate into the scratch was measured 24 and 48 h after siSNAT1-downregulation, compared to siCTR cells, using microscope IX83 (Olympus Life Science). Diameters of the scratches were analyzed using CellSens Dimension Software 1.12 (Olympus Cooperation, Tokyo, Japan).

### 2.11. Migration and Invasion Analysis with Boyden Chamber Assay

Migration and invasion of cells were analyzed using Boyden chambers containing polycarbonate filters with an 8 µm pore size (Neuro Probe Inc, Gaithersburg, MD, USA). For migration assay, a gelatin-coated filter was used and for invasion assay, Matrigel-coated filters (diluted 1:3 in DMEM without FCS; Omnilab, Bremen, Germany) were used. Fibroblast-conditioned medium was used as a chemoattractant and was filled into the lower compartment of the chamber. After typsination, 40,000 cells for migration and 200,000 cells for invasion were resuspended in DMEM without FCS and subsequently placed in the upper compartment of the chamber. After incubation for 4 h at 37 °C, the cells adhering to the lower surface of the filter were fixed, stained, and counted as described previously [25].

### 2.12. Apoptosis Analysis

For apoptosis analysis, 200,000 cells were seeded into 6-well plates. Apoptotic cells were investigated by flow cytometry using the Annexin V-FITC PromoKine Detection Kit (PromoCell GmbH, Heidelberg, Germany) according to the manufacturer’s instructions. The flow cytometry analysis was performed with a BD LSRFortessaTM X-20 cytometer (Becton Dickinson, Franklin Lakes, NJ, USA). Flow cytometry data were analyzed using FLOWJO v10 Software (BD Bioscience, Franklin Lakes, NJ, USA).

### 2.13. Cell Cycle Analysis

For cell cycle analysis, 200,000 of siCTR and siSNAT1-transfected cells were fixed and stained with propidium iodide (PI) as described previously [4]. Cytometric measurement was performed with a BD LSRFortessaTM X-20 instrument (BD Biosciences) and flow cytometry data were analyzed using FlowJo Software.

### 2.14. Senesce-Associated β-Galactosidase Staining

For senescence detection, melanoma cells were fixed and stained by using the Senescence β-Galactosidase Staining Kit (Cell Signaling Technology, Danvers, MA, USA) according to the manufacturer’s instructions. Imaging was conducted by using the microscope IX83 (Olympus Life Science). Analysis of the images was performed with the Cell Counter Plugin of Image J 1.48v software (NIH, Bethesda, MD, USA) and the percentage of senescent cells (blue) to the total cell number of cells per field of view was calculated.

### 2.15. Statistical Analysis

All experiments were performed in at least 3 independent assays. The results are shown as the mean ± standard error of the mean (SEM) calculated with the GraphPad Prism software (GraphPad Software, Inc., San Diego, CA, USA). Comparisons between groups (NHEM vs. melanoma, inhibitor MeAIB vs. control, siSNAT1 vs. siCTR) were conducted using the Student’s unpaired *t*-test. A *p*-value of <0.05 was considered statistically significant (*: *p* < 0.05).

## 3. Results

### 3.1. SNAT1 Is Upregulated In Vitro and In Vivo in Melanoma

**Figure 1:**
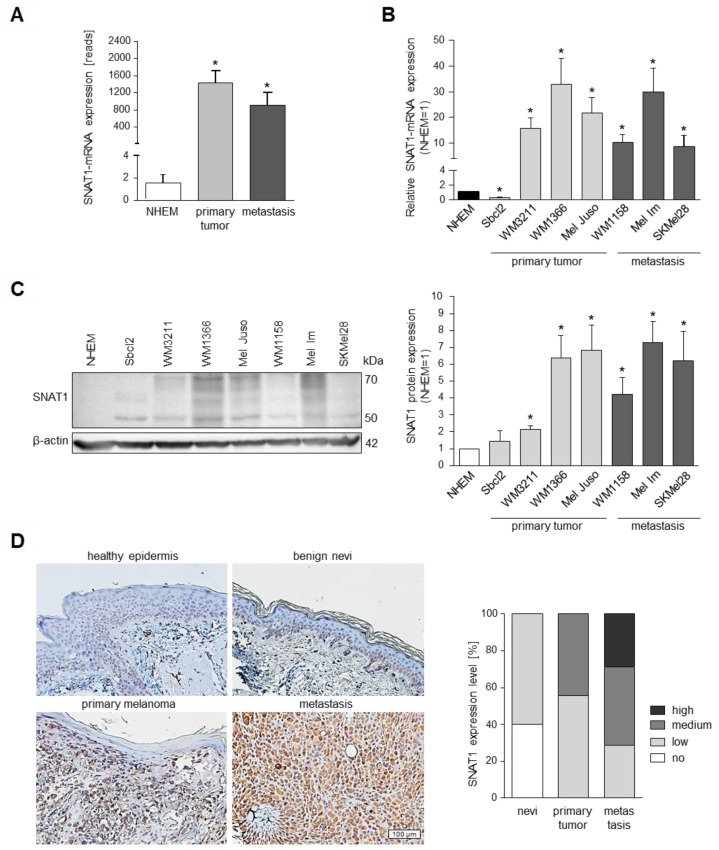
RNA and protein expression of SNAT1/SLC38A1 in different melanoma cell lines. (**A**) Mean expression from RNA-sequencing analysis of SNAT1/SLC38A1 mRNA in primary and metastatic melanoma cells in comparison to NHEM. Values represent the mean ± SEM of 2 independent experiments for NHEM and metastasis and of 6 independent experiments for primary tumor. (**B**) Confirmation of SNAT1/SLC38A1 mRNA expression with qRT-PCR of indicated melanoma cell lines obtained from primary and metastatic melanoma, normalized to β-actin and compared to NHEM. Values represent the mean ± SEM of at least 3 independent experiments. (**C**) Western blot analysis (**left**) and densitometric analysis (**right**) of SNAT1 protein level of indicated melanoma cell lines normalized to β-actin and compared to NHEM. The siPool-mediated SNAT1 downregulation confirmed that SNAT1 protein sizes range from 50–70 kDa in Western blot analysis, potentially due to glycosylation (Supplementary Figure S1). Values represent the mean ± SEM of at least 3 independent experiments. (**D**) Representative immunohistochemical staining of SNAT1 protein in healthy epidermis, benign nevi, primary, and metastatic melanoma tissue samples of formalin-fixed and paraffin-embedded tissue blocks (**left**). Percentage of no, low, medium, and high SNAT1 expression level in benign nevi (n = 10), primary (n = 9), and metastatic melanoma (n = 7) tissue IHC-staining (**right**). *p*-value < 0.05 was considered statistically significant (*). The uncropped western blot figures are presented in Supplementary Figure S5.

RNA-sequencing analysis of primary melanoma cell lines and metastatic melanoma cell lines compared to NHEMs revealed that SNAT1 expression is significantly elevated in melanoma cells (Figure 1A). To confirm the differential gene expression, we performed qRT-PCR with melanoma cell lines obtained from primary (Sbcl2, WM3211, WM1366, Mel Juso) and metastatic melanoma (WM1158, Mel Im, SKMel28), compared to NHEMs, demonstrating an upregulation of SNAT1/SLC38A1 gene expression, except for cell line Sbcl2 (Figure 1B). Investigation of SNAT1 protein expression via Western blot analysis showed an increased amount of SNAT1 protein in all melanoma cell lines when compared to NHEMs (Figure 1C), including Sbcl2. On the basis of siPool-mediated SNAT1 downregulation, we observed that SNAT1 protein includes protein sizes from 50–70 kDa in Western blot analysis (Supplementary Figure S1A).

In addition to these in vitro results, we analyzed SNAT1 expression in vivo by immunohistochemical staining of healthy epidermis, benign nevi, primary, and metastatic human melanoma tissue, and observed an elevated SNAT1 expression level in primary tumors and even higher expression in metastatic tissue (Figure 1D).

To emphasize the importance of glutamine transporters in melanoma, we investigated the gene expression of glutamine transporters SLC38A1/SNAT1, SLC38A2/SNAT2, SLC1A5/ASCT2, and SLC7A5/LAT1, which showed expression in RNA sequencing analysis in several melanoma cell lines (Supplementary Figure S2A).

### 3.2. Competitive Inhibition of SNAT1 Reduces Proliferative Potential of Melanoma Cells In Vitro

The aim of this study was to determine the functional importance of the amino acid transporter SNAT1 in human melanoma. Initially, we functionally inhibited SNAT1 with the competitive inhibitor MeAIB (Km~0.5 mM). MeAIB is an amino acid analogue, which is not metabolized and has been used extensively to study the transport function of system “A” transporters of the SNAT-family, including SNAT1/SLC38A1 [26].

**Figure 2:**
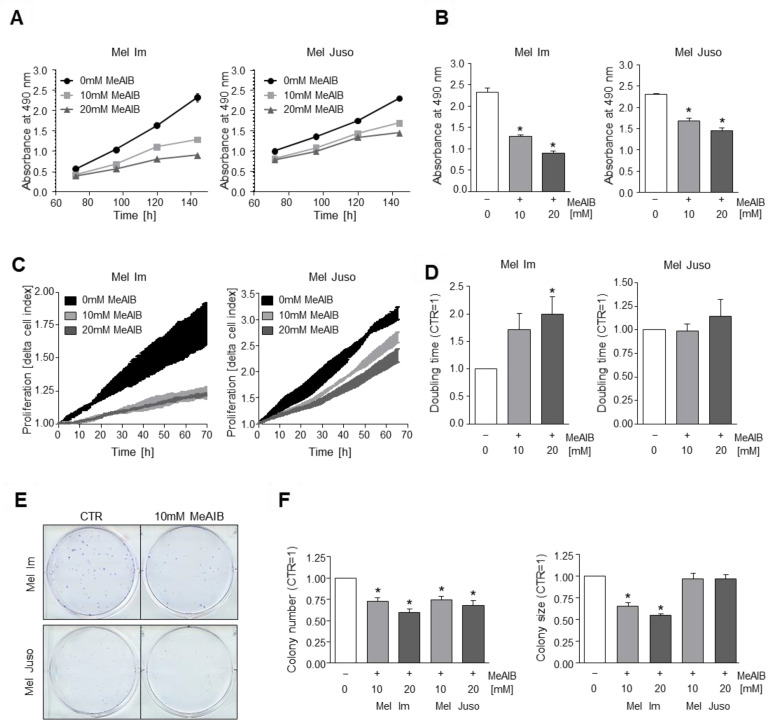
Effects of functional inhibition of SNAT1 on cell proliferation. (**A**,**B**) Proliferation analysis using XTT-assay of Mel Im (2000 cells) and Mel Juso (4000 cells) cell lines during 10 and 20 mM MeAIB-inhibitor treatment in comparison to control cells (0 mM MeAIB, equal amount of ddH20 was added). (**B**) XTT absorbance after 144 h. Values represent the mean ± SEM of 4 independent experiments. (**C**,**D**) Quantification of cell proliferation by RTCA of Mel Im and Mel Juso cell lines during 10 and 20 mM MeAIB-inhibitor treatment in comparison to control cells. (**C**) Exemplary image of RTCA curve measuring the delta cell index. (**D**) Quantified doubling time of RTCA–proliferation curve (CTR = 1). *p*-value for 10 mM MeAIB in Mel Im is 0.0746, *p*-values for 10 and 20 mM MeAIB in Mel Juso are 0.8855 and 0.4797, respectively. Values represent the mean ± SEM of 3 independent experiments. (**E**,**F**) Clonogenic assay of melanoma cell lines Mel Im and Mel Juso after 10 and 20 mM MeAIB-inhibitor treatment in comparison to control cells. Exemplary images (**E**) and quantification of colony number (**F** **left**) and colony size (**F** **right**). *p*-values for 10 mM MeAIB and 20 mM MeAIB in Mel Juso are 0.3750 and 0.2297, respectively. Values represent the mean ± SEM of 4 independent experiments. *p*-value < 0.05 was considered statistically significant (*).

We investigated the effect of functional inhibition of SNAT1 by 10 and 20 mM MeAIB, respectively, on melanoma cell growth using the XTT cell viability assay. Here, we revealed a significant reduction of proliferation in melanoma cell lines Mel Im and Mel Juso when compared to untreated control cells (Figure 2A,B). With further analysis of cell proliferation using the xCELLigence real-time cell analysis (RTCA) system, we were able to confirm these findings for melanoma cell line Mel Im (Figure 2C,D). Although having effects on both tested cell lines, the strength of effects of SNAT1 inhibition differed, suggesting a variability or heterogeneity between different tumors, which needs to be kept in mind.

Using clonogenic assays, we examined the reproductive and proliferative ability of cells to form large colonies from single cells, and revealed a significant reduction of colony number in both cell lines and a significant reduction of colony size in Mel Im (Figure 2E,F).

To evaluate alterations of glutamine transporter expression after MeAIB treatment, we determined the expression of SLC38A1/SNAT1, SLC38A2/SNAT2, SLC1A5/ASCT2, and SLC7A5/LAT1 after 10 mM MeAIB treatment for different time periods by qRT-PCR analysis. We detected a significant upregulation of these transporters, in particular in the cell line Mel Juso, in response to treatment with the inhibitor MeAIB (Supplementary Figure S2B,C). These results suggest a potential compensatory upregulation after inhibition by MeAIB, which may explain small differences in the proliferative effects of MeAIB treatment when comparing Mel Juso to Mel Im.

To evaluate the effect of MeAIB on non-malignant HEK293 cells, we conducted an XTT viability assay (Supplementary Figure S3A,B). The data revealed that MeAIB has no significant effect on HEK293 cells.

### 3.3. SNAT1 Knockdown Reduces Proliferation Rate of Melanoma Cells In Vitro

**Figure 3:**
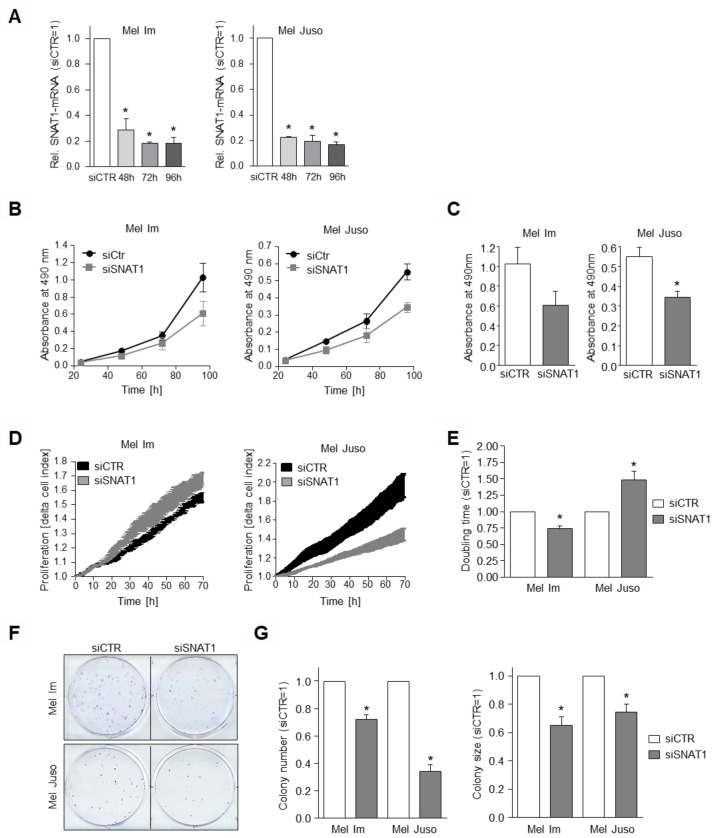
Influence of SNAT1 downregulation on cell proliferation. (**A**) Relative SNAT1 mRNA-expression normalized to β-actin after 48, 72, and 96 h siSNAT1 transfection, compared to siCTR cells of indicated melanoma cell lines. Values represent the mean ± SEM of 3 independent experiments. (**B**,**C**) Proliferation analysis using XTT assay of indicated melanoma cell lines after siSNAT1 downregulation in comparison to siCTR cells. (**B**) Exemplary absorbance at 490 nm 24, 48, 72, and 96 h after seeding siSNAT1 and siCTR cells of Mel Im and Mel Juso into the XTT assay. (**C**) Quantification of absorbance 96 h after starting the XTT assay. (Mel Im *p*-value = 0.1301.) Values represent the mean ± SEM of 3 independent experiments. (**D**,**E**) Quantification of cell proliferation with RTCA of Mel Im and Mel Juso cells after 96 h siSNAT1 transfection in comparison to siCTR cells. (**D**) Exemplary image of real time cell proliferation curve of siSNAT1 and siCTR-transfected cells. (**E**) Quantified RTCA doubling time. Values represent the mean ± SEM of at least 3 independent experiments. (**F**,**G**) Exemplary images (**F**) and quantification (**G**) of the clonogenic assay. Colony number (**G** **left**) and colony size (**G** **right**) of melanoma cell lines Mel Im and Mel Juso after siSNAT1 downregulation when compared to siCTR cells. Values represent the mean ± SEM of at least 3 independent experiments. *p*-value < 0.05 was considered statistically significant (*).

As MeAIB does not just selectively inhibit SNAT1/SLC38A1, but also SNAT2/SLC38A2 and SNAT4/SLC38A4, we further analyzed specific SNAT1 effects using an siPool for selective downregulation of SNAT1 mRNA in melanoma cells. Initially, the siRNA-mediated downregulation of SNAT1 was confirmed by mRNA (Figure 3A) and protein levels (Supplementary Figure S1). As significant downregulation of SNAT1 mRNA and protein was achieved after 96 h, we used a 96 h duration for our siRNA transfection period in our experimental design. As we observed a partly compensatory upregulation of some glutamine transporters after inhibition of the group A-amino acid transporters SNAT1/2/4 after MeAIB-inhibitor treatment, we additionally investigated the SLC38A1/SNAT1, SLC38A2/SNAT2, SLC1A5/ASCT2, and SLC7A5/LAT1 expression after siSNAT1 silencing and detected no significant effect (Supplementary Figure S2D).

To determine the effect of SNAT1 downregulation on the proliferative potential of melanoma cells, we examined proliferation rate using XTT assay and observed a significant reduction in cell line Mel Juso and a strong tendency for cell line Mel Im (Figure 3B,C).

With the RTCA system we analyzed cell proliferation and revealed a significant increase of cell index (CI) doubling time in melanoma cell line Mel Juso after siSNAT1 downregulation when compared to control cells (Figure 3D,E). Surprisingly, siSNAT1-transfected cell line Mel Im showed a significant decrease of CI doubling time (Figure 3E). Notably, the RTCA system measures impedance, which is not exclusively dependent on cell number, but also on cell morphology and changes in cell–surface adhesion. Therefore, we analyzed attachment after siSNAT1 downregulation and revealed a significantly elevated impedance after siSNAT1 silencing in cell line Mel Im (Supplementary Figure S3A), which might suggest changes in adhesion. Moreover, we investigated whether siSNAT1 downregulation has an impact on the cell size of Mel Im and determined an increase in cell size after siSNAT1 silencing (Supplementary Figure S3B), which further contributes to the elevated impedance (Figure 3E).

Additionally, colony forming potential was determined using a clonogenic assay, demonstrating a significant decrease of colony number and colony size in Mel Im and Mel Juso cells after siSNAT1 downregulation when compared to control-transfected cells (Figure 3F,G). In summary, SNAT1 downregulation showed a significant inhibitory effect on the proliferative capacity and colony forming potential of melanoma cells. In contrast, transfection of the non-malignant cell line HEK293 with siSNAT1 had no effect on proliferation (Supplementary Figure S3C).

### 3.4. SNAT1 Downregulation Leads to Reduction of Cellular Migration and Invasion

**Figure 4:**
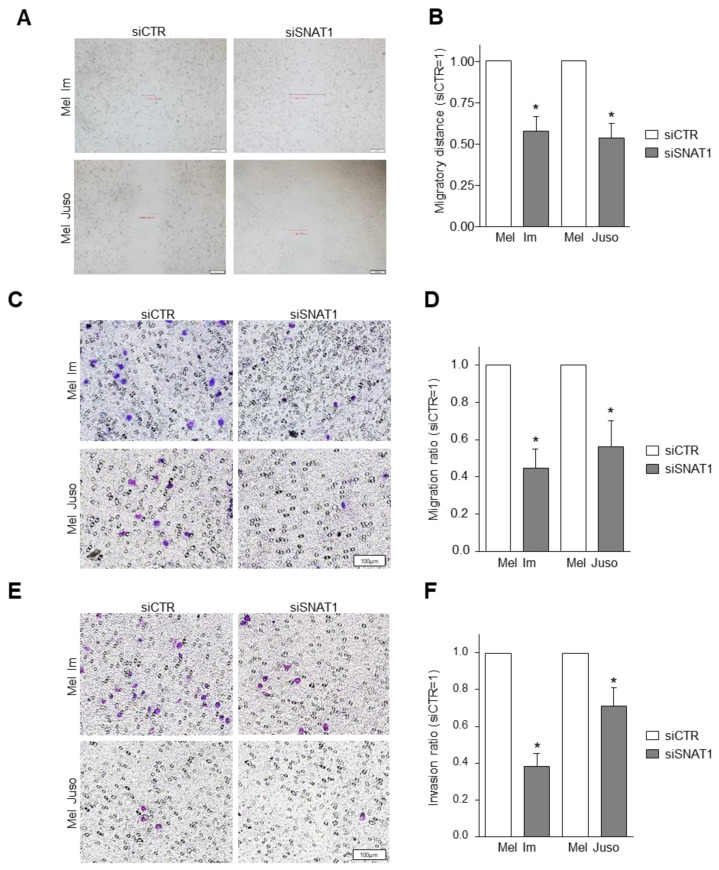
Effects of SNAT1 downregulation on migratory and invasive potential of melanoma cells. (**A**,**B**) Analysis of cell migration using scratch wound healing assay of Mel Im and Mel Juso cell lines after siSNAT1 downregulation when compared to siCTR cells. (**A**) Representative images of cell monolayer scratch of Mel Im cells 24 h and of Mel Juso cells 48 h after scratching. (**B**) Scratch width of Mel Im cells 24 h and of Mel Juso cells 48 h after scratching (siCTR = 1). Values represent the mean ± SEM of 4 independent experiments. (**C**,**D**) Analysis of cellular migration using Boyden chamber assay of Mel Im and Mel Juso cell lines after siRNA-mediated SNAT1 downregulation, compared to siCTR-transfected melanoma cells. (**C**) Representative images of Boyden chamber gelatine-coated filter membrane. (**D**) Quantification of migrated cells (siCTR = 1). Values represent the mean ± SEM of 4 independent experiments. (**E**,**F**) Analysis of cellular invasion using Boyden chamber assay of Mel Im and Mel Juso cell lines after siRNA-mediated SNAT1 downregulation, compared to siCTR-transfected melanoma cells. (**E**) Representative images of Boyden chamber Matrigel-coated filter membrane. (**F**) Quantification of invasive cells (siCTR = 1). Values represent the mean ± SEM of 4 independent experiments. *p*-value < 0.05 was considered statistically significant (*).

In further assays, we determined whether the migratory behavior of melanoma cells is influenced by SNAT1 downregulation. We examined cell migration using a wound healing assay and revealed a significantly reduced migratory capacity after siSNAT1 knockdown of both investigated cell lines Mel Im and Mel Juso (Figure 4A,B). To further evaluate cellular migration, a standardized Boyden chamber assay with gelatin-coated filters was conducted after siSNAT1 downregulation. Here, we observed a significant decrease of migrated cells of the cell lines Mel Im and Mel Juso after siSNAT1 downregulation (Figure 4C,D). Additionally, cellular invasion was assessed by Boyden chamber assay with Matrigel-coated filter membranes. We found a significant reduction of the invasive potential of Mel Im and Mel Juso after siSNAT1 downregulation when compared to control-transfected cells (Figure 4E,F), indicating that SNAT1 plays an important role in cell proliferation, migration, and invasion.

### 3.5. Reduction of SNAT1 Expression Induces Cell Cycle Arrest and Senescence in Melanoma Cells

**Figure 5:**
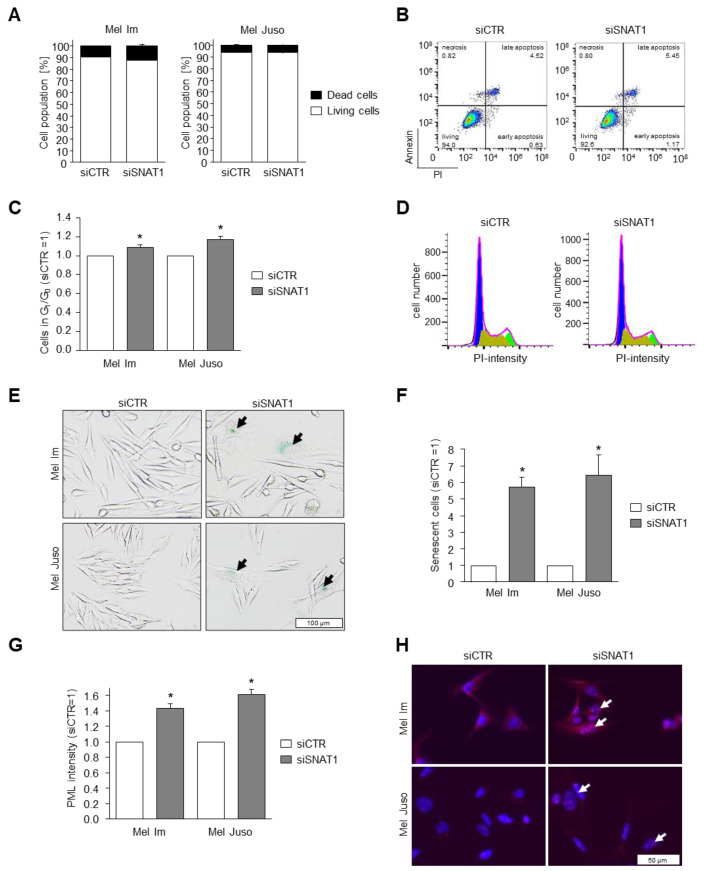
Effects of SNAT1 downregulation on apoptosis, cell cycle progression, and senescence induction. (**A**,**B**) Analysis of apoptotic and living cells using PI/ annexin-V staining examined with cytometric analysis after siRNA-mediated SNAT1 downregulation, compared to siCTR-transfected melanoma cell lines Mel Im and Mel Juso. Values represent the mean ± SEM of 3 independent experiments. (**B**) Exemplary image of flow cytometry-based apoptosis analysis of cell line Mel Im. (**C**,**D**) Flow cytometric analysis of cell cycle after siSNAT1 downregulation, compared to siCTR transfected melanoma cell lines Mel Im and Mel Juso. Values represent the mean ± SEM of 3 independent experiments. (**D**) Representative image of cytometric analysis of cell line Mel Juso. G_1_/G_0_-phase in blue, S-phase in olive green, G_2_-phase in light green. (**E**) Exemplary images of light microscopic examination of senescence-associated-β-galactosidase staining of siSNAT1 and siCTR-treated Mel Im and Mel Juso cells. Arrows indicate senescent melanoma cells. (**F**) Quantification of SA-β-galactosidase positive cells (siCTR = 1). Values represent the mean ± SEM of 3 independent experiments. (**G**,**H**) PML immunofluorescence staining of cell lines Mel Im and Mel Juso after siSNAT1 and siCTR transfection. (**G**) Quantification of PML-immunofluorescence intensity (siCTR = 1). (**H**) Representative image of PML-NB. Panels show overlay of PML (red) and DAPI (blue) staining. Arrows indicate formation of PML-NB. Values represent the mean ± SEM of 3 independent experiments. *p*-value < 0.05 was considered statistically significant (*).

To understand whether the reduced proliferation detected by RTCA and XTT analysis is caused by induction of cell death, we determined the percentage of apoptotic Mel Im and Mel Juso cells after siSNAT1-downregulation, compared to siCTR-treated melanoma cells using PI/annexin V staining and subsequent flow cytometric analysis (Figure 5A,B). We observed no increase of apoptotic cells due to SNAT1 downregulation, suggesting that reduced SNAT1 expression mainly affects the proliferative behavior of melanoma cells.

To shed light on the reduced cell growth after reduction of SNAT1 expression, we investigated the cell cycle by flow cytometry using PI to stain DNA content, and revealed a significant increase of G_1_/G_0_ phase in siSNAT1-treated cells when compared to siCTR-transfected control cells (Figure 5C,D). As we observed G_1_/G_0_ cell cycle arrest of the melanoma cells after siSNAT1 downregulation, we elucidated the cellular phenotype in more detail. For this purpose, we examined the induction of cellular senescence using senescence-associated (SA)-β-galactosidase staining. Downregulation of SNAT1 led to a significant increase of senescent melanoma cells (Figure 5E,F). The formation of promyelocytic leukemia protein nuclear bodies (PML-NB) is functionally implicated in cellular senescence in melanoma [27] and is commonly used as senescence marker [4,28]. We assessed PML-NB formation via immunofluorescence analysis and observed a significant increase of PML immunofluorescence intensity after siSNAT1 downregulation when compared to control cells (Figure 5G,H). These data indicate that a reduction of SNAT1 expression in melanoma results in reduced cell proliferation by induction of cellular senescence.

## 4. Discussion

In this study, we revealed an elevated SNAT1 expression in human primary melanoma and an even higher expression in metastatic melanoma tissue when compared to normal epidermis. Moreover, we demonstrated that SNAT1 is upregulated on the mRNA and protein level in various human melanoma cell lines obtained from primary and metastatic tumors when compared to NHEM. In our RNA sequencing analysis of the SNAT family, we found an elevated expression in comparison to NHEM only of SNAT1 and SNAT2, which emphasizes their functional importance in melanoma. Studies focusing on other cancer entities also reveal an overexpression of SNAT1 in human tumors, such as breast cancer [14], colorectal cancer [16], HCC [17], gastric cancer [18], and osteosarcoma [13], indicating an oncogenetic role of SNAT1 in cancer.

To elucidate the role of SNAT1 in human melanoma, we performed a comprehensive experimental investigation. All pharmacological inhibitors that are proposed to work for different glutamine transporters are not transporter-subtype specific [10]. Therefore, besides using the unspecific inhibitor MeAIB, we also established an siPool to downregulate SNAT1 expression, which allowed us to assess specific SNAT1 effects in melanoma cells. We demonstrated that functional inhibition using the competitive inhibitor MeAIB and siRNA targeting SNAT1 attenuated cell proliferation as determined by XTT, RTCA, and clonogenic assay. These findings are in accordance with previous studies which revealed that SNAT1 promotes proliferation and tumor growth in colorectal cancer [16], breast cancer [14], osteosarcoma [13], and gastric cancer [18].

Interestingly, in the RTCA assay, we observed a decrease of CI doubling time and enhanced attachment after SNAT1 silencing of the cell line Mel Im. A decrease of doubling time and increase of cell attachment can be caused by changes of cell morphology, in particular, enlargement of cell bodies, which represents a sign for cellular senescence [29]. In previous studies, we demonstrated that especially the melanoma cell line Mel Im exhibits an enlargement of cell bodies and extension of cellular processes during senescence [4]. Here, we confirmed an induction of senescence after SNAT1 downregulation by G_1_/G_0_ cell cycle arrest, SA-β-galactosidase staining, and PML-NB formation in both cell lines. However, until today no direct link between SNAT1 and senescence has been demonstrated. One study showed that treatment of AML cells with the tyrosine kinase inhibitor gilteritinib reduces the expression of several proteins, including SNAT1, which is associated with an increased SA-β-galactosidase activity [30]. It is not clear which cellular mechanism induced by gilteritinib leads to the induction of senescence, nonetheless, this study supports our finding that the amino acid transporter SNAT1 is connected to the repression of senescence in tumor cells.

Reduced transport of glutamine and leucine by inhibition of ASCT2 in melanoma cells leads to decreased mTOR signaling, resulting in cell cycle arrest [31]. Therefore, we hypothesize that cell cycle progression of melanoma is promoted by signaling pathways that are activated by SNAT1-transported amino acids. Another possible mechanism by which SNAT1 regulates senescence might involve ROS, which is a critical mediator of senescence [32,33]. Reduced transport of glutamine by silencing of SNAT1 could lead to decreased synthesis of glutathione, resulting in elevated ROS levels and induction of cellular senescence. In melanoma, the role of glutamine and glutathione in senescence remains to be elucidated, but our proposed mechanism is consistent with previous studies. In pancreatic ductal adenocarcinoma and breast cancer, glutamine deprivation leads to elevated ROS levels, thereby inducing senescence [34,35].

Breast cancer cells that were able to escape from therapy-induced senescence exhibit upregulation of the glutamine transporter ASCT2 and, to smaller extent, also of SNAT1 and SNAT2. In addition, glutamine starvation of several cancer types suppresses escape from therapy-induced senescence [36], promoting the assumption that there is a direct connection between glutamine transport and thereby enhanced glutamine metabolism and senescence in cancer cells.

The increase of 10–20% of cells arrested in G_1_/G_0_ phase after knockdown of SNAT1 expression, as assessed by cell cycle analysis, does not completely explain the great extent of reduced proliferative and colony forming capacity of siSNAT1-transfected melanoma cells. This finding indicates that melanoma cells respond heterogeneously to SNAT1 silencing. This assumption is supported by the fact that melanoma is a highly heterogenous tumor. Here, melanoma cells are able to shift between different transcriptional programs, cell cycle states and cellular phenotypes to adapt to various exogenous effects [37]. A phenomenon that might explain why the population of cells arrested in G_1_/G_0_ phase is only moderately increased when compared to the greater effect on proliferation might be the development of slow-cycling melanoma cells due to amino acid depletion. Subpopulations of slow-cycling tumor cells were described in some cancer types, including melanoma [38,39,40,41]. Studies showed that slow-cycling JARID1B^high^ melanoma cells maintain a continuous tumor growth, are resistant to various chemotherapeutic drugs [37,42], and are involved in tumor recurrence [41].

Based on our findings, the reduction in cell growth and colony formation capacity after SNAT1 silencing are caused by a combinatory effect of reduced viability, by impaired metabolism due to amino acid depletion, and by an elevated induction of cellular senescence.

Besides a reduction of cell growth, SNAT1 silencing also decreased the cell migration and invasion rate of melanoma cells, suggesting a role of SNAT1 during melanoma progression and the formation of metastases. These data are in accordance with other cancers, where SNAT1 overexpression is associated with increased proliferation rate, tumor size, tumor invasion, and migration [13,14,16,18,19].

Our data suggest that inhibition of SNAT1 can be a promising therapeutic option in treating melanoma. However, the choice of the way of inhibition will be crucial, as the inhibitor MeAIB showed cell line-dependent effects, whereas the knockdown resulted in strong effects in all cell lines.

Our data provide novel evidence that SNAT1 plays an essential role in melanoma development and progression by promoting cell proliferation, colony formation, migration, and invasion, but also by inhibiting cellular senescence. Moreover, SNAT1 overexpression is associated with a poorer prognosis of patients with breast cancer [14], osteosarcoma [13], AML [19], and gastric cancer [18], hinting at SNAT1 as a potential drug target for cancer therapy. Therefore, the development of pharmacological inhibitors that selectively and effectively target SNAT1 will be a fruitful work program for the future.

## Figures and Tables

**Table 1:** Overview of used cell lines.

Cell Line	Origin	Mutation
NHEM	Healthy tissue	-
Sbcl2	Primary tumor (radial growth phase)	N-RASQ61K
WM3211	Primary tumor (vertical growth phase)	p53T724G
WM1366	Primary tumor (vertical growth phase)	N-RASQ61L
Mel Juso	Primary tumor	N-RASQ61L
WM1158	Metastasis	BRAFV600E, PTENDel/V343E
Mel Im	Metastasis	BRAFV600E
SKMel28	Metastasis	BRAFV600E, PTENT167A, CDK4R24C, p53L145R
HEK293	Embryonic kidney	-

**Table 2:** Primers for qRT-PCR.

Primer	Forward Primer 5′-3′	Reverse Primer 5′-3′	Annealing Temperature	Measurement Temperature
SLC38A1/ SNAT1	GCTTTGGTTAAAGAGCGGGC	CTGAGGGTCA-CGAATCGGAG	60 °C	78 °C
SLC38A2/ SNAT2	CTGAAGACGTCTGCGTGAGA	CCAAGGATTCCACTGCCCAC	60 °C	86 °C
SLC1A5/ ASCT2	CTGGCTGGTAACCGCTACTC	TGTCCGAAAGCTGGGAGTTC	60 °C	86 °C
SLC7A5/ LAT1	GGCCGAGGAGAAGGAAGAGG	CCTCCAGCATGTAGGCGTAG	60 °C	86 °C

## Data Availability

The data presented in this study are available on request from the corresponding author.

## Supplementary Materials

The following are available online at https://www.mdpi.com/article/10.3390/cancers14092151/s1, Figure S1: Downregulation of SNAT1 on the protein level, Figure S2: Expression of glutamine transporters in melanoma, Figure S3: Effects of MeAIB and siSNAT1 on HEK293 cell line. Figure S4: Attachment and cell size of melanoma cells after downregulation of SNAT1. Figure S5: Uncropped Western blots.
