# Supplementary material for: Role of Amino Acid Transporter SNAT1/SLC38A1 in Human Melanoma
Source: Cancers (Basel). 2022 Apr 26;14(9):2151. doi: 10.3390/cancers14092151 (PMC9099705; doi:10.3390/cancers14092151)
Supplement: Supplementary file 1 [file cancers-14-02151-s001.zip › cancers-1670426-supplementary.pdf]

cancers

MDPI

Supplementary Materials

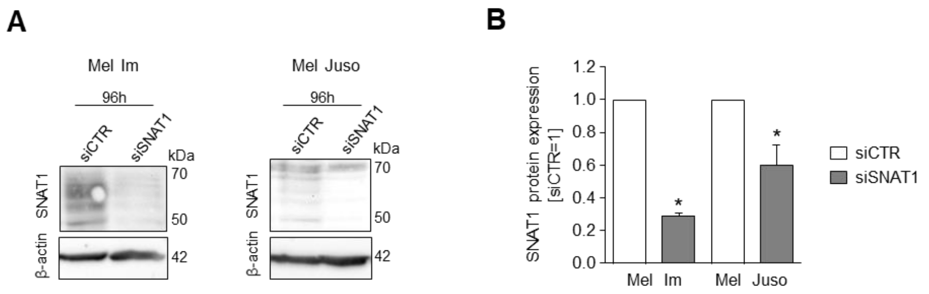

Supplement Figure S1. Downregulation of SNAT1 on the protein level. **(A)** Exemplarily Western blot of SNAT1 and β-actin protein after 96h siSNAT1 transfection compared to siCTR of indicated mel-anoma cell lines. **(B)** Relative SNAT1 protein-expression normalized to β-actin after 96h siSNAT1 transfection compared to siCTR of indicated melanoma cell lines. Values represent the mean ± SEM of 3 independent experiments, p-value < 0.05 was considered statistically significant.

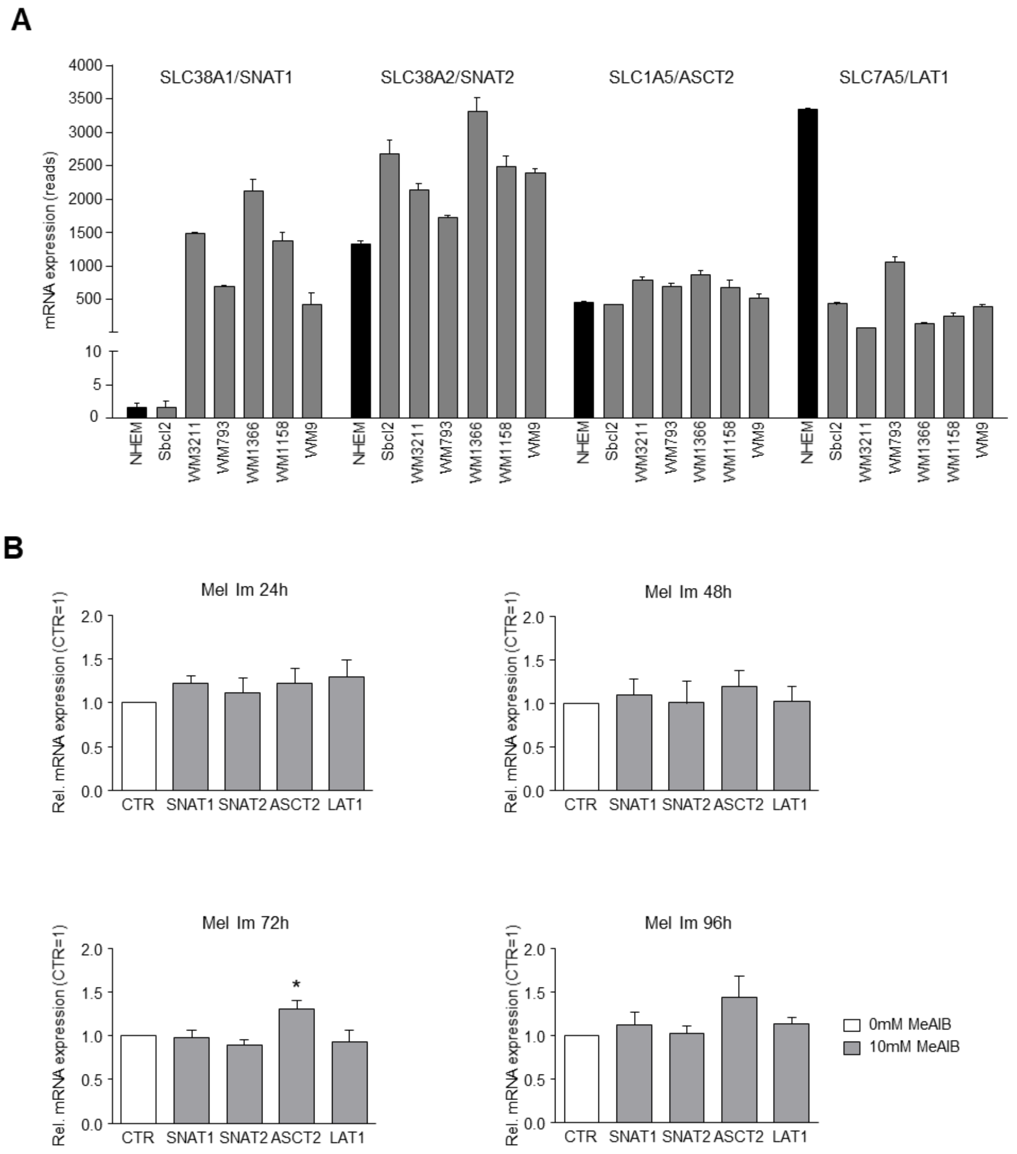

C

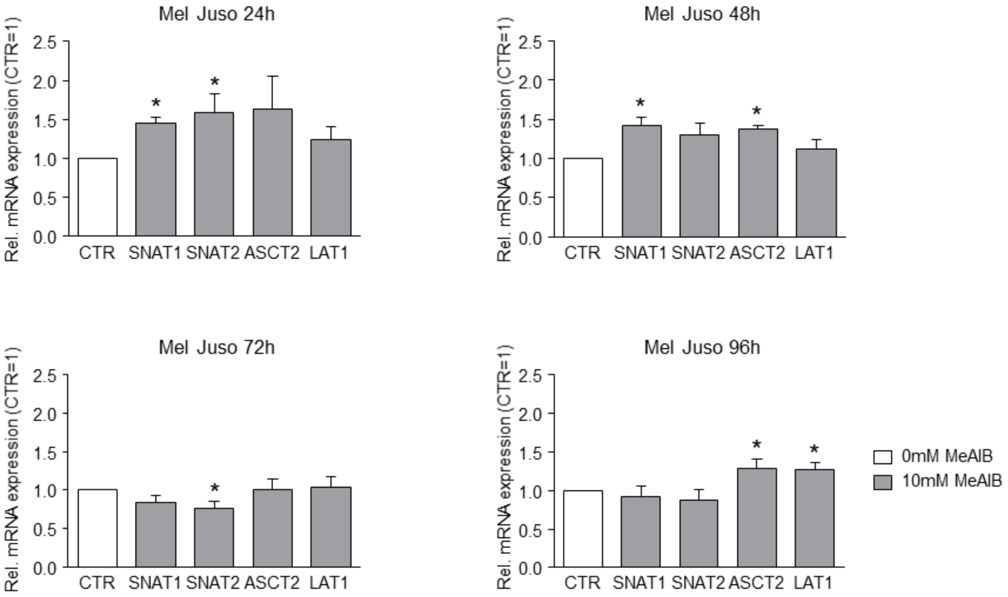

D

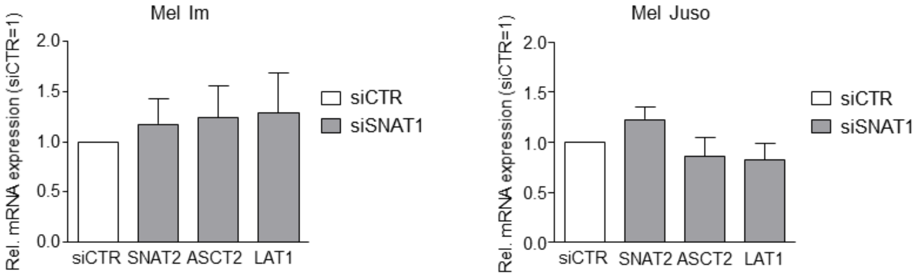

Supplement Figure S2. Expression of glutamine transporters in melanoma. **(A)** Mean mRNA expres-sion from RNA-sequencing analysis of SLC38A1/SNAT1, SLC38A2/SNAT2, SLC1A5/ ASCT2, and SLC7A5/LAT1 of indicated melanoma cell lines in comparison to NHEM. Values represent the mean ± SEM of 2 independent experiments. (**B) and (C)** Analysis of mRNA expression of SLC38A1/SNAT1, SLC38A2/SNAT2, SLC1A5/ASCT2 and SLC7A5/LAT1 using qRT-PCR of cell lines Mel Im and Mel Juso after MeAIB-inhibitor treatment (10mM MeAIB) for 24h, 48h, 72h and 96h normalized to β-actin in comparison to untreated control cells (0mM MeAIB). Values represent the mean ± SEM of 4 independent experiments. **(D)** Analysis of mRNA expression of SLC38A2/SNAT2, SLC1A5/ASCT2 and SLC7A5/LAT1 using qRT-PCR of cell lines Mel Im and Mel Juso after silencing of SNAT1 with siSNAT1 for 96h in comparison to siCTR cells. Values represent the mean ± SEM of 4 independent experiments, p-value <0.05 was considered statistically signifi-cant.

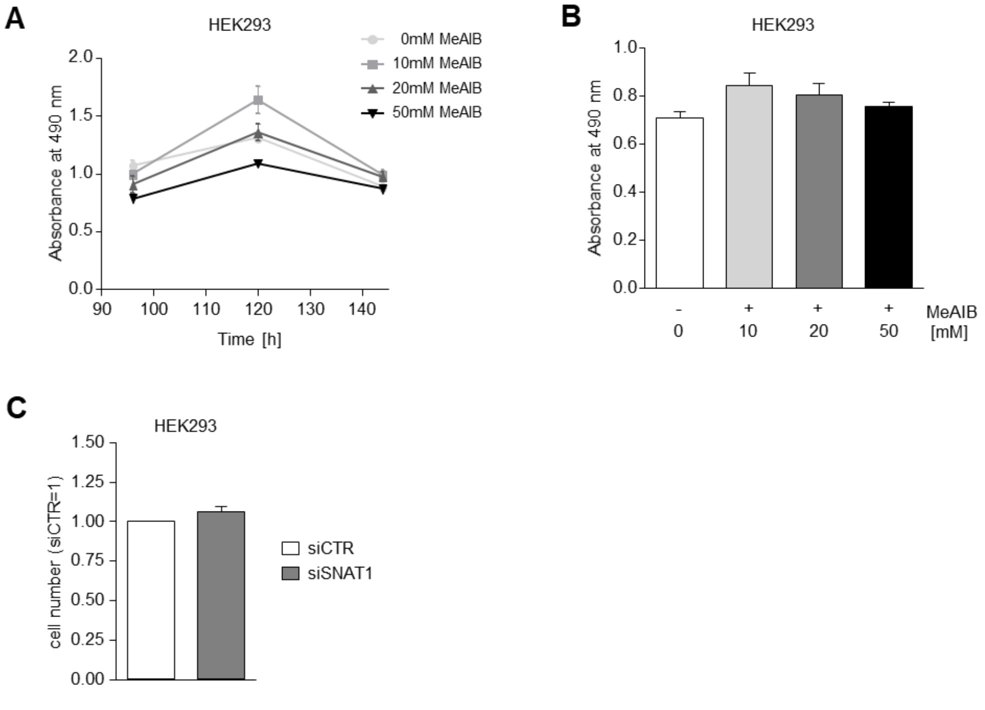

Supplement Figure S3. Effects of MeAIB and siSNAT1 on HEK293 cell line. **(A) and (B)** Proliferation analysis using XTT-assay of HEK293 cell line (4000 cells) during 10mM, 20mM and 50mM MeAIB-inhibitor treatment in comparison to control cells. **(A)** Exemplary absorbance at 490nm 96h, 120h and 144h after starting the assay. **(B)** Quantification of absorbance 144h after starting the assay. Val-ues represent the mean ± SEM of 3 independent experiments, p-value of <0.05 was considered sta-tistically significant. **(C)** Analysis of cell number of siSNAT1 transfected HEK293 cells. HEK293 were transfected with siSNAT1 for 96h and subsequently counted. Values represent the mean ± SEM of 3 independent experiments, p-value of <0.05 was considered statistically significant.

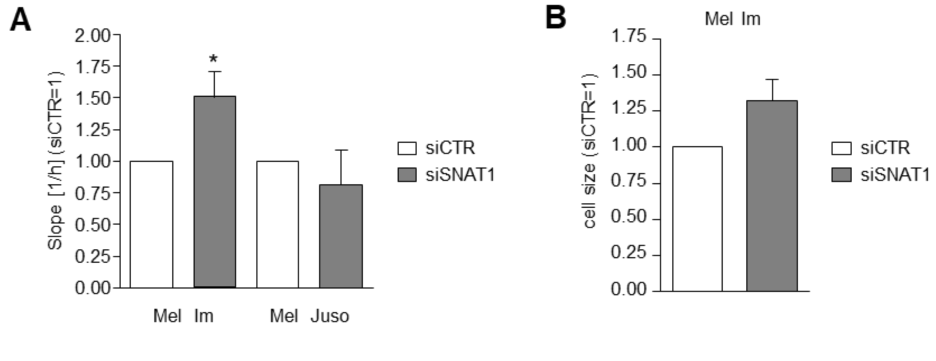

Supplement Figure S4. Attachment and cell size of melanoma cells after downregulation of SNAT1. **(A)** Quantification of cell attachment with RTCA of Mel Im (2.5h) and Mel Juso (4h) cell line after siSNAT1 silencing in comparison to siCTR cells. Values represent the mean ± SEM of 3 independent experiments. **(B)** Quantification of cell size of Mel Im siSNAT1 in comparison to siCTR cells was analyzed using immunofluorescence staining for F-actin. Values represent the mean ± SEM of 3 independent experiments, p-value <0.05 was considered statistically significant.

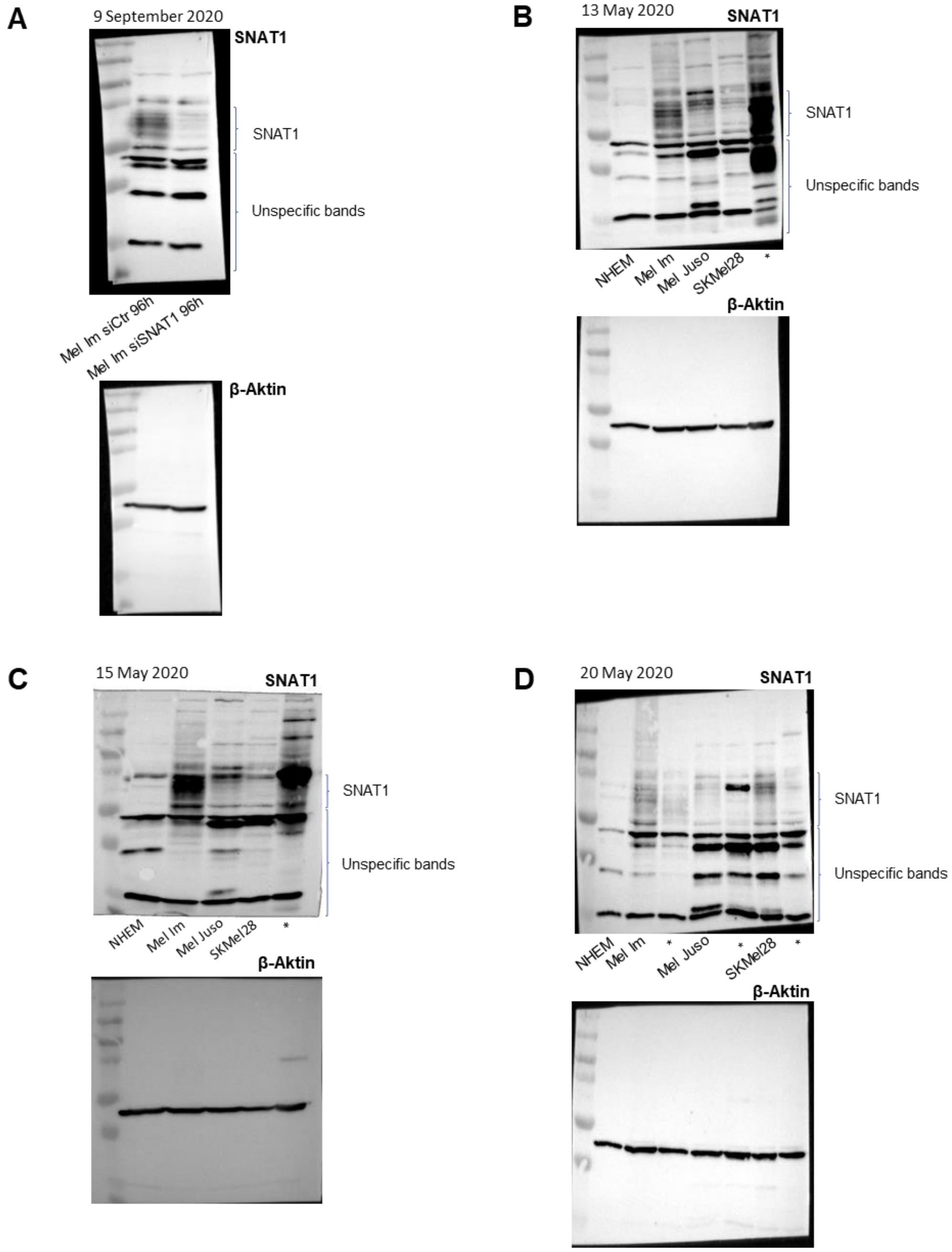
A
9 September 2020
SNAT1
SNAT1
Unspecific bands
Mel Im siCtr 96h
Mel Im siSNAT1 96h
β-Aktin
B
13 May 2020
SNAT1
SNAT1
Unspecific bands
NHEM
Mel Im
Mel Juso
SKMel28
*
β-Aktin
C
15 May 2020
SNAT1
SNAT1
Unspecific bands
NHEM
Mel Im
Mel Juso
SKMel28
*
β-Aktin
D
20 May 2020
SNAT1
SNAT1
Unspecific bands
NHEM
Mel Im
*
Mel Juso
*
SKMel28
*
β-Aktin

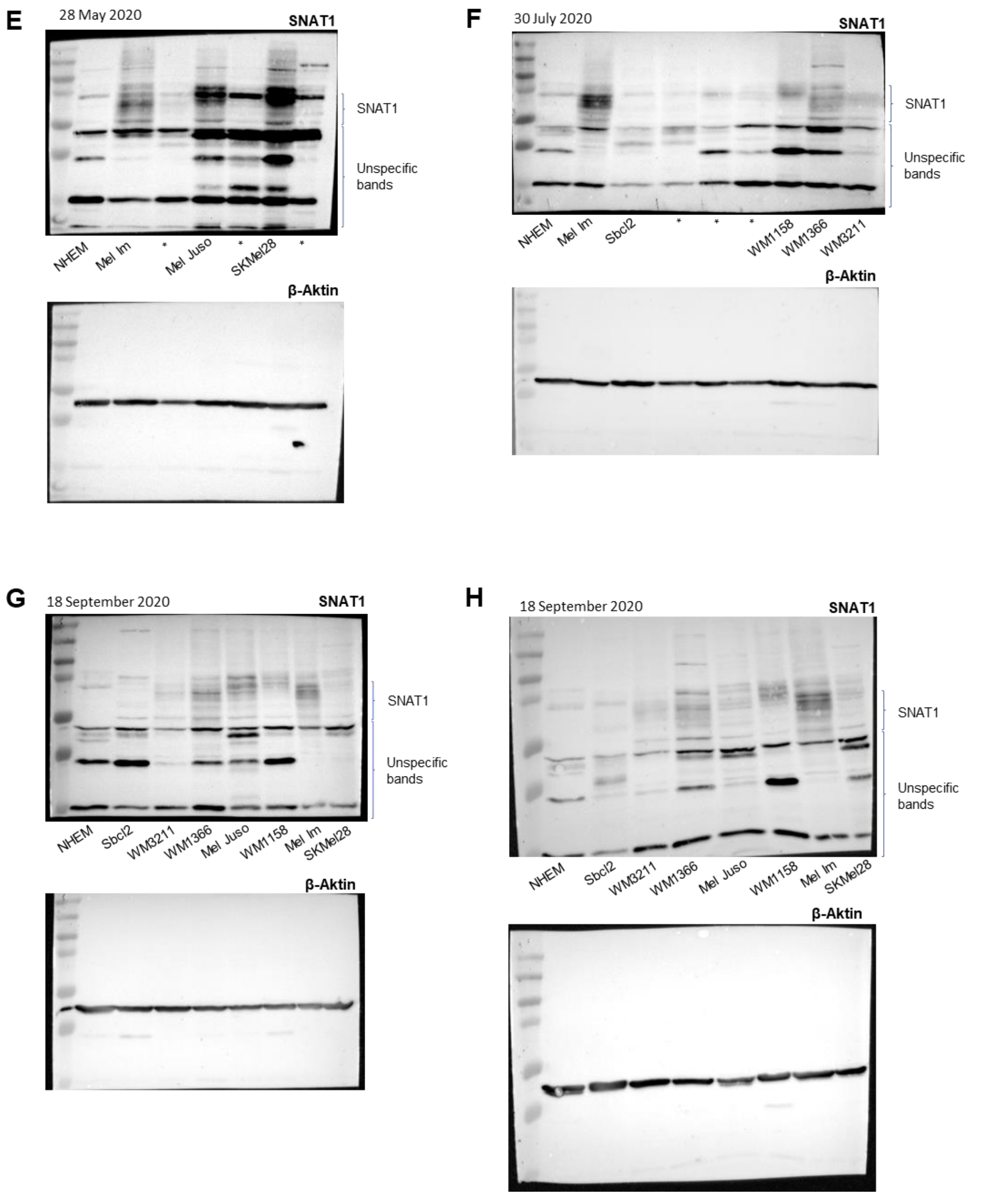
E
28 May 2020
SNAT1
SNAT1
Unspecific bands
NHEM
Mel Im
*
Mel Juso
*
SKMel28
*
β-Aktin
F
30 July 2020
SNAT1
SNAT1
Unspecific bands
NHEM
Mel Im
Sbcl2
*
*
*
WM1158
WM1366
WM3211
β-Aktin
G
18 September 2020
SNAT1
SNAT1
Unspecific bands
NHEM
Sbcl2
WM3211
WM1366
Mel Juso
WM1158
Mel Im
SKMel28
β-Aktin
H
18 September 2020
SNAT1
SNAT1
Unspecific bands
NHEM
Sbcl2
WM3211
WM1366
Mel Juso
WM1158
Mel Im
SKMel28
β-Aktin

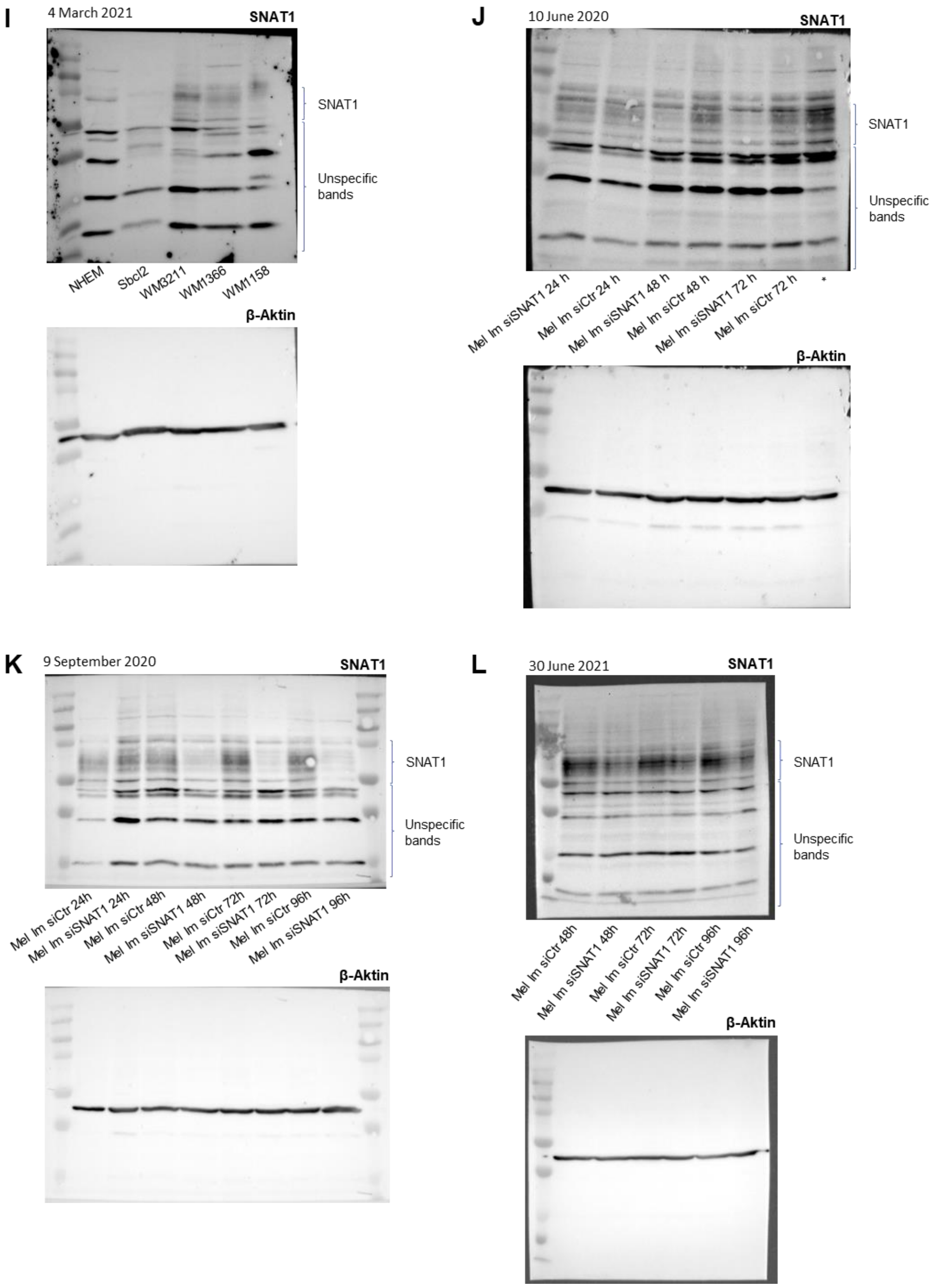
I
4 March 2021
SNAT1
SNAT1
Unspecific bands
NHEM
Sbcl2
WM3211
WM1366
WM1158
β-Aktin
J
10 June 2020
SNAT1
SNAT1
Unspecific bands
Mel Im siSNAT1 24 h
Mel Im siCtr 24 h
Mel Im siSNAT1 48 h
Mel Im siCtr 48 h
Mel Im siSNAT1 72 h
Mel Im siCtr 72 h
*
β-Aktin
K
9 September 2020
SNAT1
SNAT1
Unspecific bands
Mel Im siCtr 24h
Mel Im siSNAT1 24h
Mel Im siCtr 48h
Mel Im siSNAT1 48h
Mel Im siCtr 72h
Mel Im siSNAT1 72h
Mel Im siCtr 96h
Mel Im siSNAT1 96h
β-Aktin
L
30 June 2021
SNAT1
SNAT1
Unspecific bands
Mel Im siCtr 48h
Mel Im siSNAT1 48h
Mel Im siCtr 72h
Mel Im siSNAT1 72h
Mel Im siCtr 96h
Mel Im siSNAT1 96h
β-Aktin

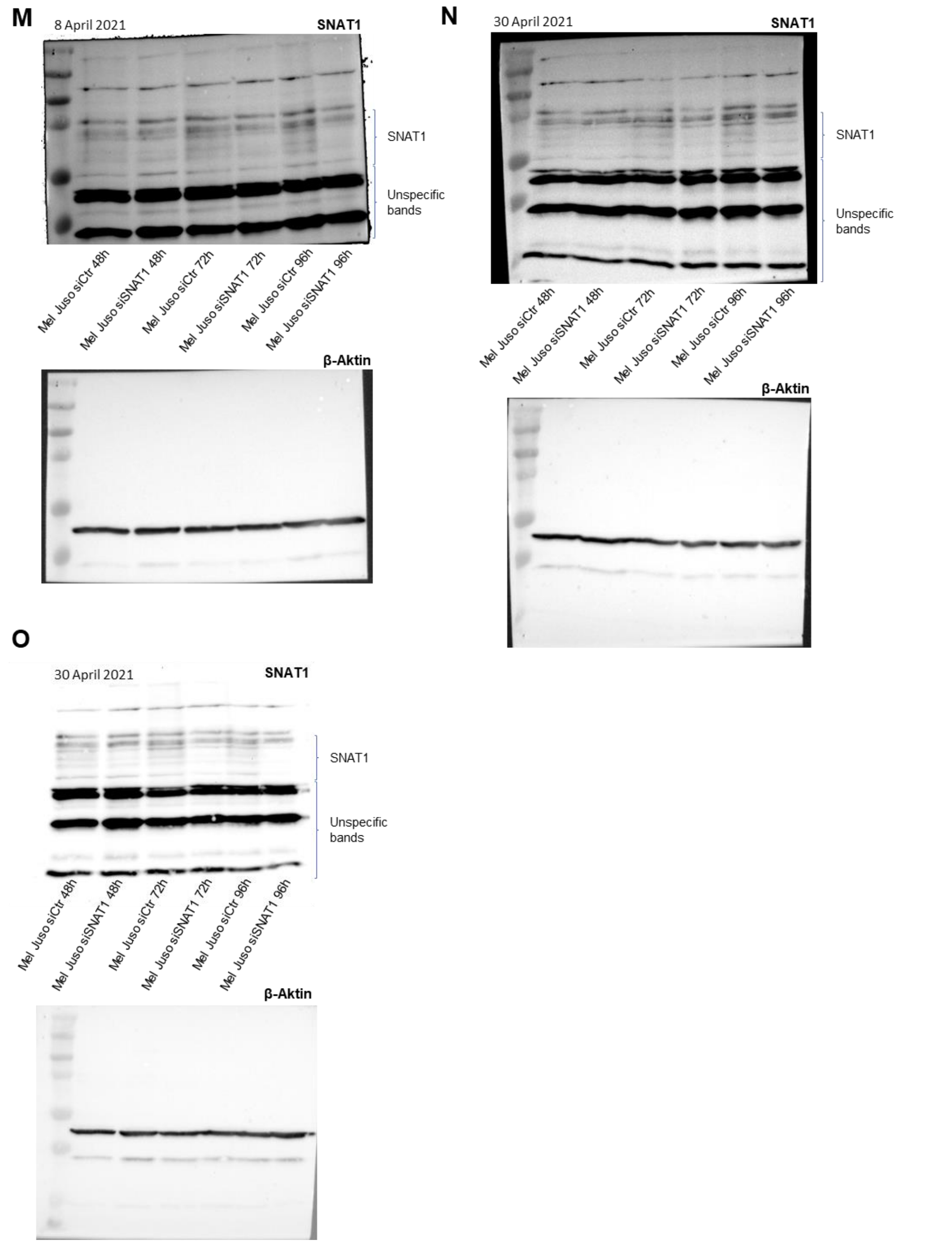

Supplementary Figure S5. Uncropped Western Blots. **(A)** siRNA proof for antibody specificity. This blot exhibits several bands per lane. Downregulation of SNAT1 expression by transfection with an siPool reveals that only bands between 50 and 70kDa are regulated by siSNAT1. This shows that these bands are specific for SNAT1. The other bands, however, are due to unspecific binding of the antibody that was used. **(B)-(I)** Western Blot analysis of SNAT1 and β-Aktin protein level of indi-cated melanoma cell lines. Associated with Figure 1C. **(J)-(L)+(A)** Western Blot analysis of SNAT1 and β-Aktin protein level of melanoma cell line Mel Im for establishing siSNAT1 mediated knock-down of SNAT1 expression. Associated with Supplementary Figure 1A. In our paper, we only show the values for transfection for 96h. Blots also contain 24h, 48h and 72h because we first had to estab-lish the siPool mediated knockdown of SNAT1. **(M)-(O)** Western Blot analysis of SNAT1 and β-Aktin protein level of melanoma cell line Mel Juso for establishing siSNAT1 mediated knockdown of SNAT1 expression. Associated with Supplementary Figure 1A. In our paper, we only show the values for transfection for 96h. Blots also contain 48h and 72h because we first had to establish the siPool mediated knockdown of SNAT1. Lanes labeled with an asterisk (*) contain samples that are unrelevant for this study. Dates indicate time of development of the blots.
